# Reconfigurable ferroelectric chiral nanostructures enable fast-switchable optical spatial differentiation

**DOI:** 10.1038/s41377-026-02363-w

**Published:** 2026-06-26

**Authors:** Wen Chen, Dong Zhu, Su-Nan Chen, Yi-Heng Zhang, Si-Jia Liu, Rui Sun, Yi-Ming Wang, Lin Zhu, Shi-Hui Ding, Shi-Jun Ge, Yan-Qing Lu, Peng Chen

**Affiliations:** National Laboratory of Solid State Microstructures, Key Laboratory of Intelligent Optical Sensing and Manipulation, College of Engineering and Applied Sciences, and Collaborative Innovation Center of Advanced Microstructures, Nanjing University, Nanjing, 210093 China

**Keywords:** Nanophotonics and plasmonics, Liquid crystals

## Abstract

Analog spatial differentiation is an emerging computational paradigm. By virtue of high speed and low-power consumption, optical method plays an important role in data compression, microscopy and computer vision. However, most developed optical differentiators are static and lack the reconfigurability of differentiation functions. Herein, we propose a reconfigurable space-variant ferroelectric chiral nanostructure to dynamically control the optical differentiation. Via switching the polarity of external electric field, 1st-order/2nd-order spatial differentiation or bright-field imaging can be actively selected with an ultra-short response time down to 62 μs. Edges of biological cells, as well as intensity objects, can be well identified, while their direct imaging is also achievable synchronously. Such fast-switchable differentiator shows excellent reliability and reversibility for over 1.8 million cycles and over 200 days. This work advances the ingenious building of ferroelectric nanostructures, and offers an important glimpse into their potential in neuromorphic photonics, biomedical microscopy and artificial intelligence.

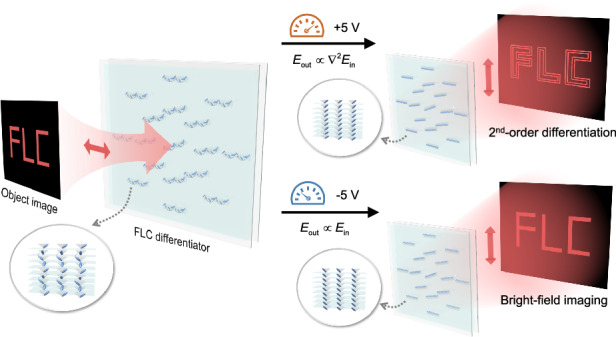

## Abstract (web summary)

We propose a reconfigurable space-variant ferroelectric chiral nanostructure to dynamically control the optical differentiation. Via switching the polarity of external electric field, spatial differentiation or bright-field imaging can be actively selected with an ultra-short response time. This work advances the ingenious building of ferroelectric nanostructures, and offers an important glimpse into their potential in neuromorphic photonics, biomedical microscopy and artificial intelligence.

## Introduction

Liquid crystals (LCs)^1–3^, featured by both anisotropy and high fluidity, are usually self-assembly with a specific alignment tendency. This gives rise to the birefringence of LCs and sensitive response to external stimuli such as heat, electric/magnetic field and light irradiation^4^. Recently, LCs have been widely used in a myriad of applications, from the most famous LC displays^5–7^ to the emerging structured light manipulation^4,8–11^. Typically, LCs can be categorized into distinct mesophases including nematic, cholesteric and smectic LCs^1,2,4^. As one of the most attracting LCs, smectic C* phase ferroelectric LCs (FLCs)^12,13^ show intriguing chiral and ferroelectric nature. The FLC molecules are arranged to behave like two-dimensional liquid in layers, which establishes a spatial periodicity in the direction perpendicular to the layers^1,12,13^. Moreover, the FLC molecule tilt spirals around the normal from layer to layer, with a fixed tilt angle and the rotated azimuthal angle. Notably, triggered by proper external electric field, the ferroelectric nature leads to the fast reconfigurability of FLC molecule orientation, i.e., equivalent optical axis. The tunability and the rapidity of FLCs facilitate enormous possibilities in various areas, such as virtual/artificial reality, beam steering and hologram^14–20^. These unique properties may also respond to the call of contemporary optical analog computing.

Optical analog computation catches eyes due to its distinguished advantages of high parallelism, fast speed and low consumption^21–27^. Compared to traditional ways, it offers more potential in numerous communities, such as autonomous driving, biomedical imaging and defect inspection^28–30^. In particular, optical spatial differentiation has been highly sought-after as an essential mathematical operation to extract edges, the most significant features in images, for real-time recognition or segregation of target objects^31,32^. Various nanophotonic differentiators have been demonstrated to realize edge detection, such as metasurfaces^33–42^, LCs^43,44^ and photonic chips^45^. Nonetheless, so far, most developed devices have been restricted to a static function and lack the dynamic reconfigurability of optical differentiation. Indeed, more flexibility, more sustainability and more hands-on feasibility imminently are the evolutionary trajectory of versatile optical computing operators^22^. In this regard, FLCs’ impressive reconfigurability and fast response exactly make it a competitive candidate for dynamic and on-demand differentiation in optical computing.

**Fig. 1:**
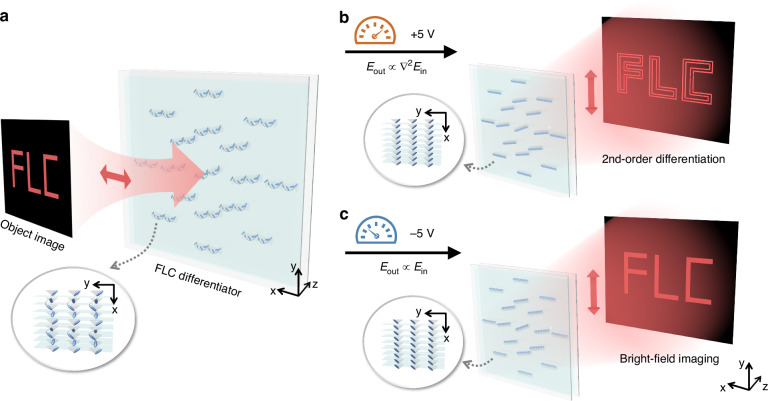
Schematic illustration of the fast-switchable optical spatial differentiation based on a reconfigurable FLC chiral nanostructure. **a** The FLC chiral nanostructure without external electric field, inset: the local cross-sectional diagram. **b** 2nd-order differentiation under +5 V, inset: the local cross-sectional diagram of the helix-suppressed FLC nanostructure, with the molecules rotating to one side. **c** Bright-field imaging under −5 V, inset: the local cross-sectional diagram of the helix-suppressed FLC nanostructure, with the molecules rotating the other side. The blue ellipsoid represents the unit director of FLC. The gray cone denotes the helical cone. The blue plane represents the smectic layer of FLC. The gray spring illustrates the chiral structure. The red double-ended arrow designates the polarization direction of the polarizer or analyzer

In this work, we propose a reconfigurable space-variant ferroelectric chiral nanostructure for the polychromatic and ultra-fast switchable optical spatial differentiation (Fig. 1). Such chiral lamellar nanostructure of FLCs is directed by the delicate photopatterned layer and self-assembles into the inhomogeneously-variant optical axis. Under opposite polarities of the applied electric field, the overall optical axis rotates synchronously, contributing to the active switch between spatial differentiation and bright-field imaging. 1st-order and 2nd-order FLC differentiators are designed to realize fast tunable imaging between boundary auto-identification/fine-positioning and bright-field imaging, respectively. After over 1.8 million cycles and 200 days, the FLC nanostructures remain good performance with the ultra-fast switching time around 62 μs. For transparent objects, the rapid dynamic identification of the fine edges of biological cells can also be achieved, indicating great potential for biomedical applications. This work promotes the hierarchical construction of ferroelectric nanostructures, develops advanced materials for dynamic light manipulation, and offers a new insight for high-frame-rate intelligent optical information processing.

## Results

### Reconfigurable ferroelectric chiral nanostructures

Ferroelectricity in LCs brings marvelous dynamics. The FLC chiral nanostructure naturally augments the capability as a reconfigurable active optical operator. Here, we propose a heliconical architecture based on smectic C* FLCs, to enable artificially switching between desired processing, i.e., spatial differentiation and bright-field imaging. As vividly shown in Fig. 1a, rod-like FLC molecules constrained within the cell self-assemble into helical twisting lamellar structure under the planar boundary conditions. The spontaneous polarization vector *P*_S_ is always perpendicular to the normal of the smectic layer, and simultaneously rotates along with the lying helix. The response of polarization to an applied electric field results in molecules’ rotation motion (Fig. 1b, c) due to the electrically suppressed helix (ESH) mode^46,47^. The equivalent local optical axis also rotates. The proper electric field perpendicular to the helix axis completely suppresses the helix at *V* ≥ *V*_th_ (*V*_th_ is the threshold voltage), and furthermore the polarity of the applied electric field determines the exact rotational direction ( + *θ* or –*θ*, *θ* is the tilt angle, i.e., half of the cone angle of the FLC helical structure) along the surface of the helical cone.

**Fig. 2:**
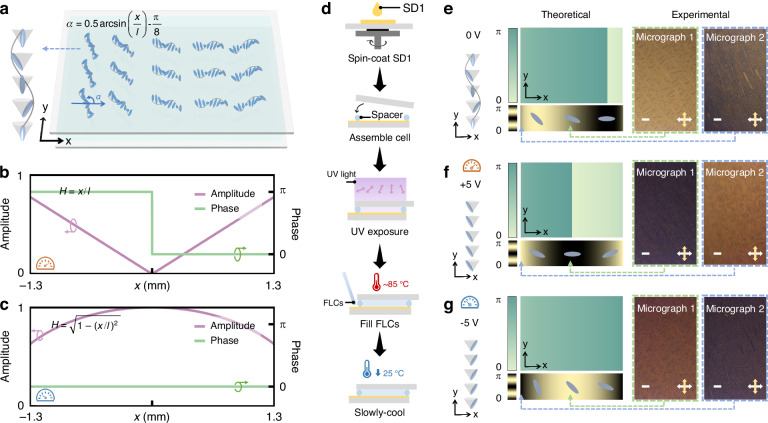
Illustration, fabrication and characterization of the reconfigurable FLC chiral nanostructure for dynamic 1st-order differentiation. **a** Dynamic 1st-order FLC differentiator. The blue ellipsoid represents the unit director of FLC molecule. The gray spring and the gray helical cone illustrate the chiral structure. **b** The amplitude and the phase of the theoretical transfer function *H*(*x*,*y*) under +5 V. **c** The amplitude and the phase of the theoretical transfer function *H*(*x*,*y*) under −5 V. **d** Fabrication process of the FLC differentiator. The purple arrow represents the local linear polarization distribution of UV light. **e**–**g** The theoretical distribution of the optical axis (upper left), simulated (lower left) and experimental optical micrographs (middle and right) of the fabricated FLC differentiator under crossed polarizers (**e**) without external electric field, (**f**) under positive electric field ( + 5 V), and (**g**) under negative electric field (−5 V), respectively. The color bar of simulated micrographs illustrates the optical axis distribution of FLC differentiator. The hazy-blue ellipsoid represents the local equivalent optical axis. The white and yellow double-ended arrows label the direction of the polarizer and analyzer, respectively. All scale bars are 100 μm

To construct the FLC chiral lamellar structure, we exploit UV photopatterning technique^4,9^ with azo-dye SD1 coated on one substrate (Sample Fabrication in Methods and Supplementary Note 1). Figure 2d demonstrates the whole fabrication process of the FLC chiral nanostructure. Firstly, sulfonic azo-dye SD1 was spin-coated on one of the precleaned bare indium tin oxide (ITO)-coated glass plates. Subsequently, 1.5-μm-diameter spacers, as pillars supporting the sandwich-like configuration, were used with UV glue to assemble the empty cell. After the multi-step photopatterning, the SD1 molecules were aligned perpendicular to the illuminated UV polarization, and constructed a space-variant alignment as expected. Thus, the pre-designed pattern was encoded into the empty cell. Finally, the FLC materials were filled into the prepared cell at 85 °C (over the clearing point), and gradually cooled to room temperature at a rate of 0.1 °C/min. The asymmetric planar boundary conditions^48^ and slow cooling process can drastically reduce the defects within the formed FLC chiral lamellar structure.

### Dynamically switchable boundary auto-identification

1st-order differentiation^49^ is one of the most significant foundations in edge extraction and feature classification. Under the combined effect of crossed polarizers, the output *E*_out_(*x*,*y*) of a 4 *f* system should be expressed as ${E}_{{\rm{out}}}(x,y)\propto { {\mathcal F} }\{{ {\mathcal F} }\{{E}_{{\rm{in}}}(x,y)\}\cdot H(\lambda fx,\lambda fy)\}$. Here, *λ* is the wavelength, *f* is the focal length of lens, and *H*(*x*,*y*) represents the transfer function of the proposed FLC differentiator at the Fourier plane (i.e., the confocal plane of the 4 *f* system). The transfer function *H*(*x*,*y*) can be calculated as

1 $$\begin{array}{ll}H(x,y) & =\left[\begin{array}{cc}0 & 1\end{array}\right]\left[\begin{array}{cc}\cos (2\alpha (x,y)) & \sin (2\alpha (x,y))\\ \sin (2\alpha (x,y)) & -\,\cos (2\alpha (x,y))\end{array}\right]\left[\begin{array}{l}1\\ 0\end{array}\right]\\ & =\sin (2\alpha (x,y))\end{array}$$

*α*(*x*,*y*) is the orientation angle of the local optical axis with respect to the *x*-coordinate. Therefore, *H*(*x*,*y*) is highly relative to *α*(*x*,*y*). Accordingly, to achieve ${E}_{{\rm{out}}}(x,y)\propto \frac{\partial }{\partial x}{E}_{{\rm{in}}}(x,y)$ (i.e., 1st-order differentiation) and ${E}_{{\rm{out}}}(x,y)\propto {E}_{{\rm{in}}}(x,y)$ (i.e., bright-field imaging) synchronously, the distribution of *α*(*x*,*y*) is delicately designed, which is formulated as

2 $$\alpha (x,y)=0{\rm{.5arcsin}}(x/l)-\beta +{\alpha }_{0}$$

Here, *β* is chosen as the tilt angle *θ* = π/8 (π/8 is the ideal value), *α*_0_ is the initial angle and *l* equals half the sample size along the *x*-coordinate. Figure 2a exhibits a distinctive space-variant pattern encoded into the FLC chiral structure. The corresponding transfer function is written by

3 $$H(x,y)=\left\{\begin{array}{ll}x/l & {\alpha }_{0}=+\theta \\ \sqrt{1-{(x/l)}^{2}} & {\alpha }_{0}=-\theta \end{array}\right.$$

If the polarity of the applied electric field is positive, *α*_0_ = +*θ* = π/8 and *H*(*x*,*y*) = *x/l*. Thus, the amplitude of *H*(*x*,*y*) increases linearly with |*x/l* | (the purple line in Fig. 2b), and the phase of *H*(*x*,*y*) is shifted by π at *x/l* = 0 (the green line in Fig. 2b). This reflects the nature of 1st-order spatial differentiation (i.e., ${E}_{{\rm{out}}}(x,y)\propto \frac{\partial }{\partial x}{E}_{{\rm{in}}}(x,y)$). It extracts high-frequency components (e.g., edges) and restrains low-frequency components in the target incident image. When altering the polarity, *α*_0_ = -*θ* = -π/8 and $H(x,y)=\sqrt{1-{(x/l)}^{2}}$. Considering the practical condition of *x/l* « 1, *H*(*x*,*y*) is approximately believed to be 1, which indicates the input light will transmit without obvious change. The corresponding amplitude and phase of *H*(*x*,*y*) are respectively shown in the Fig. 2c, respectively. These verify that the proposed FLC differentiator can perform the 1st-order spatial differentiation and bright-field imaging synchronously just by electrical control, making it a useful tool for diverse optical processing tasks.

The theoretical FLC optical axis distributions under different electric fields are respectively illustrated in the upper left column of Fig. 2e–g. The external electric field is responsible for the +*θ*/−*θ* rotation of the overall optical axis. The simulated micrographs under crossed polarizers are displayed beneath each theoretical schematic, respectively, revealing a congruence with the theoretical predictions. The remarkable difference of the optical axis distribution is well verified by the variant brightness distribution. In addition, local experimental micrographs (middle and right columns of Fig. 2e–g) of two distinct regions (edge and center) are exhibited, wherein the changed distribution of the local optical axis is further implied under different external electric fields. Nevertheless, the defect lines in all micrographs remain unchanged, owing to the fixed lamellar structures determined by the photoalignment patterns.

**Fig. 3:**
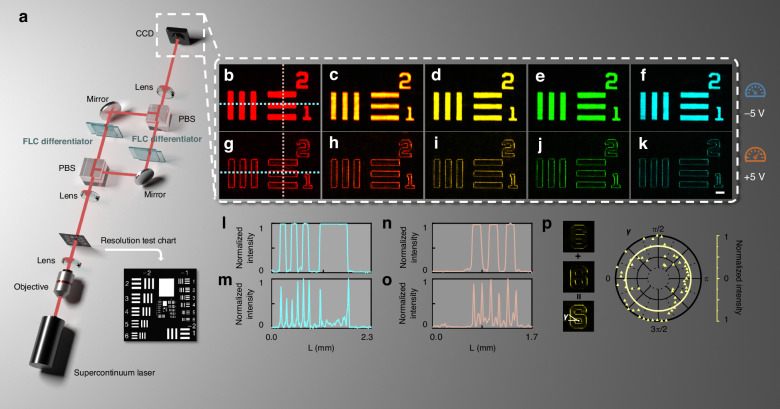
Dynamically switchable 1st-order optical spatial differentiation. **a** Experimental setup of optical spatial differentiation via the reconfigurable FLC chiral nanostructure. Inset: 1951 United States Air Force (USAF) resolution test chart. **b**–**f** The bright-field images under −5 V and **g**–**k** the 1st-order edge images under +5 V at 630 nm, 600 nm, 580 nm, 550 nm, 490 nm, respectively. **l**, **n** are corresponding intensity analysis of blue and light-pink dotted lines marked in (**b**), respectively. **m**, **o** are corresponding intensity analysis of blue and light-pink dotted lines marked in (**g**), respectively. **p** The two-dimensional intensity analysis of the closed line in number ‘6’. *γ* is the polar angle. Maximum intensities are identified radially for each *γ* (at 3° intervals). The radial distance represents the normalized intensity. The average of all measured maximum intensities over the full circumference, denoted by yellow line, is 0.76. The scale bar is 200 μm

The dynamic switch between 1st-order differentiation and bright-field imaging via the fabricated FLC differentiator is investigated by the optical setup in Fig. 3a. An expanded light illuminates the object (1951 USAF resolution test chart) set at the front focus of the 4 *f* system. A configuration of Mach-Zehnder interferometer is used to intergate these two one-dimentional differentiation (see details in Methods). The CCD detects complete two-dimentional edge images at the back focus of the 4 *f* system. When the polarity of the applied electric field is negative, the bright-field images (Fig. 3b–f) are obtained because of *H*(*x*,*y*) approximately 1 as expected. With the positive electric field applied, the FLC differentiator in each arm performs one-dimentional 1st-order differentiation along *x*/*y* coordinate (Supplementary Fig. 1 and left column of Fig. 3p), respectively. Thanks to the interferometer, Fig. 3g–k show the enhanced two-dimentional edge images (${|{E}_{{\rm{out}}}(x,y)|}^{2}\propto {|\frac{\partial }{\partial x}{E}_{{\rm{in}}}(x,y)|}^{2}+{|\frac{\partial }{\partial y}{E}_{{\rm{in}}}(x,y)|}^{2}$) at wide working wavelengths of 630 nm, 600 nm, 580 nm, 550 nm, and 490 nm, respectively. The normalized intensity distributions (Fig. 3m, o) along two crossed directions of Fig. 3g are vividly presented to compare with those of Fig. 3b at negative electric field (Fig. 3l, n), which quantitatively verifies the high-contrast and high-quality edge detection. Specifically, the intensity distribution of the closed line in the detection result of number ‘6’ (Fig. 3p) is also measured, which matches the conceptual two-dimensional design well.

### Ultra-fast response and excellent stability

**Fig. 4:**
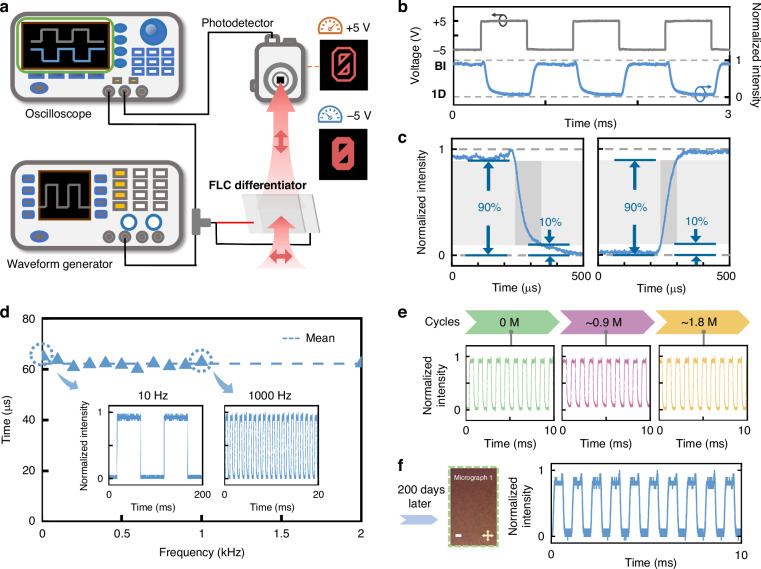
Ultra-fast response and excellent stability of the proposed FLC differentiator. **a** Schematic of response measurement. Red double-ended arrows represent the polarizer and analyzer, respectively. **b** Response curve under alternating-current square wave signal of 10 Vpp and 1 kHz. BI: bright-field imaging. 1D: 1st-order differentiation. **c** Response time of bright-field imaging to differentiation and differentiation to bright-field imaging, respectively. **d** Response time versus the frequency of applied signal. The dash line demonstrates the mean value 62 µs under 10 Vpp within the frequency range up to 2 kHz. Inset: response curve at 10 Hz and 1 kHz, respectively. **e** Analysis of switching cycles at 10 Vpp and 1 kHz after operating 0 cycles, 0.9 million cycles and 1.8 million cycles. **f** The polarized optical micrograph (middle region of the sample, at −5 V) and analysis of switching cycles at 10 Vpp and 1 kHz after 200 days. The white and yellow double-ended arrows label the direction of the polarizer and analyzer, respectively. The scale bar is 100 μm

The exceptional electro-optical properties of ESH-FLCs allow an ultra-fast switching speed in the dynamic differentiation system. Measured by the setup in Fig. 4a, the intensity response to external electric field is presented in Fig. 4b. The switching time is obtained by recording the intensity change (the duration between 10 and 90%) of the output under the applied square wave signal. For 10 Vpp and 1 kHz, it is as short as 79 μs (bright-field imaging to 1st-order differentiation, left panel of Fig. 4c) and 55 μs (1st-order differentiation to bright-field imaging, right panel of Fig. 4c), respectively. The oscillating pattern of output intensity in response to the regular applied signal suggests that this differentiation system can modulate light rapidly and efficiently. This is highly desirable in real-time optical computing and information processing. Moreover, the differentiation maintains an exceptional frequency stability. The average response exhibits little fluctuation around 62 μs below 2 kHz (Fig. 4d). The detailed switching time from bright-field imaging to 1st-order differentiation and reverse process are shown in Supplementary Fig. 2. The insets of Fig. 4d demonstrate two good response curves at 10 Hz and 1 kHz, respectively. The intensity under cyclic voltage is highly repeatable for over 1.8 million cycles (Fig. 4e). Following continuous operation, the response curve remains similar, indicating its stable capability for efficient and fast response. The fabricated FLC differentiator also shows very impressive long-term durability, which is revealed by the stable cycles measured after 200 days (Fig. 4f).

### Dynamically switchable boundary fine-positioning

**Fig. 5:**
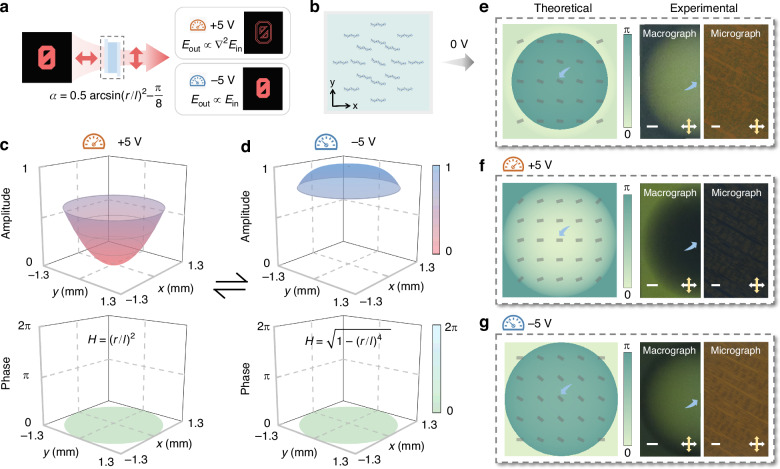
Illustration and characterization of the reconfigurable FLC chiral nanostructure for dynamic 2nd-order differentiation. **a** Conceptual illustration of the switchable 2nd-order differentiation. Red double-ended arrows represent the polarizer and analyzer, respectively. **b** Dynamic 2nd-order FLC differentiator. The blue ellipsoid represents the unit director of FLC molecules. The gray spring and the gray helical cone illustrate the chiral structure. **c** The amplitude and phase of the transfer function *H*(*x*,*y*) under +5 V. **d** The amplitude and phase of the transfer function *H*(*x*,*y*) under −5 V. **e**–**g** The theoretical distribution of the optical axis, macrographs of the fabricated FLC differentiator and optical micrographs of the center region under crossed polarizers (**e**) without external electric field, (**f**) under positive electric field (+ 5 V), and (**g**) under negative electric field (−5 V), respectively. The gray rod represents the local equivalent optical axis. White and yellow double-ended arrows label the direction of the polarizer and analyzer, respectively. The scale bars of macrographs and micrographs are 500 μm and 100 μm, respectively

2nd-order differentiation^49,50^ excels in edge localization and precise edge detection (e.g., detecting roof edge) compared with 1st-order differentiation. By working in coordination with bright-field imaging, it is expected to obtain more accurate details simultaneously with the original image. As conceptually illustrated in Fig. 5a, both on-demand operations can be realized by regulating the polarity of external electric field applied on the proposed FLC differentiator. Here, the designed orientation angle *α* of the equivalent optical axis follows:

4 $$\alpha (r)=0{\rm{.5arcsin}}[(r/l{)}^{2}]-\beta +{\alpha }_{0}$$

where $r=\sqrt{{x}^{2}+{y}^{2}}$, *β* is chosen as *θ* = π/8, and *l* equals half the sample size along the *x/y*-coordinate. Similarly, the working transfer function now can be derived as

5 $$H(r)=\left\{\begin{array}{ll}{(r/l)}^{2} & {\alpha }_{0}=+\theta \\ \sqrt{1-{(r/l)}^{4}} & {\alpha }_{0}=-\theta \end{array}\right.$$

If under the positive electric field, the overall optical axis rotates +*θ* (i.e., *α*_0_ = +*θ*). The amplitude of *H*(*r*) is proportional to (*r*/*l*)^2^, while the phase maintains constant (Fig. 5c). Accordingly, the output optical field is ${E}_{{\rm{out}}}(x,y)\propto {\nabla }^{2}{E}_{{\rm{in}}}(x,y)$ (${\nabla }^{2}=\frac{{\partial }^{2}}{\partial {x}^{2}}+\frac{{\partial }^{2}}{\partial {y}^{2}}$), indicating the two-dimensional 2nd-order spatial differentiation. Conversely, if the polarity flips, *α*_0_ will take the opposite value -*θ*. Given that *r*/*l* « 1, *H*(*r*) can be mathematically expressed as 1 under approximation (Fig. 5d). In this case, ${E}_{{\rm{out}}}(x,y)\propto {E}_{{\rm{in}}}(x,y)$. Corresponding simulation results are shown in Supplementary Fig. 3. In all, the FLC superstructure alternatively performs either two-dimensional 2nd-order differentiation or bright-field imaging on the incident light, depending on the polarity of applied electric field.

Through the high-resolution photopatterning technology, a space-variant FLC ferroelectric nanostructure (Fig. 5b) is demonstrated as theory. The theoretical optical axis (the left column of Fig. 5e) without applying electric field presents a radial distribution. The continuous brightness changings in the macrograph (the middle column of Fig. 5e) and micrograph (center region of the sample, the right column of Fig. 5e) verify the theoretical distribution. The overall optical axis distribution rotates by +*θ* (Fig. 5f) or −*θ* (Fig. 5g) under a positive or negative electric field, respectively. This rotation is further confirmed by comparing the macrographs and micrographs (center region of the sample) presented in Fig. 5f, g.

**Fig. 6:**
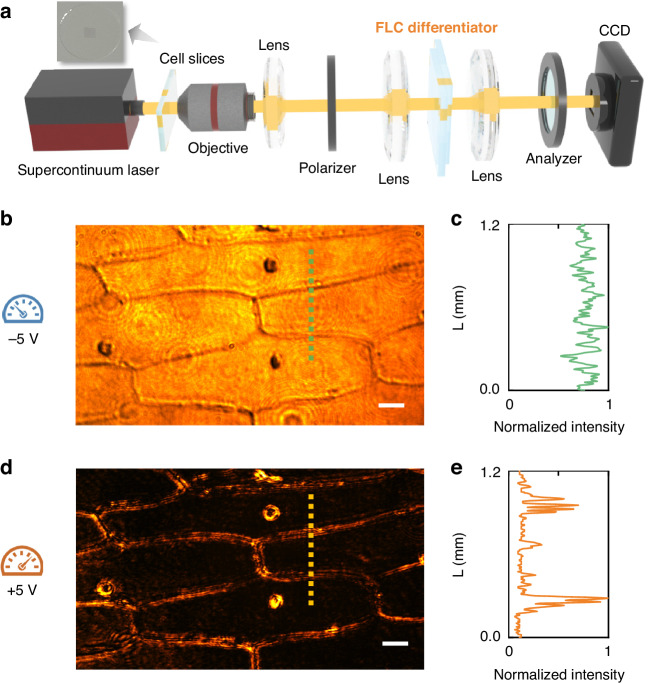
Dynamically switchable 2nd-order optical differentiation for precise edge extraction of biological cells. **a** Experimental setup of 2nd-order optical spatial differentiation via the reconfigurable FLC chiral nanostructure. Inset: unstained onion epidermal cells. **b** The bright-field images under −5 V and **d** the 2nd-order edge images under +5 V at 600 nm. **c**, **e** are corresponding intensity analysis of green and orange dotted lines marked in (**b**, **d**), respectively. All scale bars are 200 μm

For the intensity objects, the proposed FLC differentiator is not only available to detect polychromatic two-dimensional 2nd-order edges (Supplementary Fig. 4f–j), but also capable of bright-field imaging (Supplementary Fig. 4a–e) by electrical control. Ideally, the response time is dependent on the applied electric field (*E* = *V*/*d*) (Supplementary Fig. 5). Therefore, the thickness should be optimized to obtain a compromise between working energy consumption, response time and imaging quality (Supplementary Note 2 and Supplementary Fig. 6). Moreover, the phase objects imaging is especially crucial in biology and material science, as it can reveal more detailed information about the structure and composition of transparent objects, i.e., biological cells. For accurate edge extraction of those cells, Fig. 6a shows the used setup, which is mainly composed of a modified 4 *f* system and crossed polarizers. If applying a negative electric field, the bright-field images of unstained onion epidermal cells are obtained in a broad band (Fig. 6b and Supplementary Fig. 7a–e). If changing the polarity, the FLC chiral structure identifies the two-dimensional 2nd-order edges (Fig. 6d and Supplementary Fig. 7f–j). Corresponding intensity analyses (Fig. 6c, e) verify the outstanding capability both for precise edge detection and direct imaging, which can greatly facilitate the fine observation of cell morphology and behavioral patterns in biological researches.

## Discussion

The proposed FLC heliconical superstructure offers a marvelous platform to spur the optical information processing onto a more intelligent and real-time stage. The reconfigurable optical axis under opposite electric field provides convenient operation, tunable functionalities, and high maneuverability. Once fabricated, the FLC differentiator exhibits robust performance against humidity fluctuations and is stable over a large temperature range (Supplementary Figs. 8 and 9) in a wide band (Supplementary Fig. 10). Compared with existing optical differentiators^37,42,44,51–55^, our scheme provides comparable functional diversity, working band and resolution, while exhibits much faster, more energy saving and cheaper (Supplementary Tables 1 and 2). Furthermore, the system complexity is anticipated to be further reduced by fully exploiting spin-orbit interactions^37,56^, integrating lens phase^56,57^ or leveraging heterogeneous integration^37,58^. Unlike non-volatile platforms such as phase-change materials^59^ (e.g., Ge_2_Sb_2_Te_5_^55^ or Sb₂S₃^42^), our device requires a sustaining electric field. Fortunately, the necessary field is exceptionally low in magnitude, and such a minimal power consumption can be readily supplied by common cells. Moreover, most previous optical differentiators, especially the commonly used polarization gratings (Supplementary Fig. 11), employ the finite difference to approximate differential operations. In contrast, our proposed FLC differentiator is exactly derived from the desired analytical solution. Notably, here, the absence of any approximation-related errors results in an improved resolution during the edge detection (Supplementary Fig. 12).

The optionality for either spatial differentiation or bright-field imaging efficiently provides distinct and comprehensive morphological information of target objects. Besides, this FLC differentiator can be easily incorporated into conventional imaging systems (e.g., microscopes)^31^, and its ultra-fast response makes the synchronous observation possible. The temporal resolution enables the multi-modal analysis of dynamic processes such as cellular events. It promises for the visualization of live object and accentuation of regional boundaries in biology and diagnostics. High speed is essential to minimize the time gap between sequentially captured images, effectively treating them as a simultaneous, multimode snapshot. The FLC differentiator’s high-frame-rate is compatible with commercial high-speed imagers, which carries a foreshadowing for real-time acquisition and computation with a larger field of view and less crosstalk.

In summary, we propose a new strategy for fast-switchable optical differentiation via the delicate FLC chiral nanostructure. Through asymmetric surface-anchored photopatterning technique, FLC with lying helix self-assembles into a space-variant chiral lamellar structure. On-demand 1st-order/2nd-order differentiation or bright-field imaging can be efficiently realized for target intensity/phase objects. By switching the polarity of external electric field, an ultra-fast response time is obtained down to 62 μs. It shows excellent reliability and reversibility for over 1.8 million cycles and over 200 days. This work explores the potential of ferroelectric nanostructures for real-time image processing and analog computing, and discloses their unprecedented possibilities in the fields of neuromorphic photonics, machine vision and bio-microscopy.

## Materials and methods

### Materials

The sulfonic azo-dye SD1 is dissolved in dimethylformamide at a concentration of 0.35 wt% as a photoalignment agent. The SD1 molecules tend to reorient perpendicular to the linear polarization direction of the illuminated UV light. FLC material (BEAM Co., USA) with *P*_0_ = 245 nm, *P*_S_ = 110 nC/cm^2^ and *θ* = 25° near ideal 22.5° is chosen. Its phase transition of Isotropic → SmA* → SmC* is at 78 °C and 72 °C, respectively.

### Sample fabrication

One bare ITO-coated glass substrate (1.5 × 2.0 cm^2^) was subjected to ultrasonic cleaning and UV-Ozone treatment. Then, it was embedded with the photoalignment layer of SD1 by spin-coating, and cured at 100 °C for 10 min sequentially. Another clear bare ITO-coated glass substrate was used to seal the cell with spacer-doped UV glue. The sandwich-like configuration was formed with desired thickness of 1.5 μm. The pre-designed pattern was memorized in the SD1 layer through the multi-step polarized exposure with the digital-micromirror-device (DMD)-based photopatterning system^4,9^. SD1 molecules tend to reorient perpendicular to the illuminated UV polarization direction. The DMD (Discovery 3000, Texas Instruments) is consisted of 1920 × 1080 micromirrors with the single pixel size of 10.8 μm × 10.8 μm, and a 2× objective was used. The FLC materials were injected into the photopatterned cell at 85 °C, and then gradually cooled from isotropic phase to smectic C* phase at a rate of 0.1 °C/min. The FLC helix axis tend to self-assemble parallel to the SD1 molecules whose distribution is following the designed pattern. (More details can be seen in Supplementary Note 1)

### Characterizations

Temperature control was conducted by a hot stage (LTS120, Linkam, UK). All micrographs were recorded by a polarized optical microscope (Ci-POL, Nikon, Japan) with crossed polarizer and analyzer under the transmission mode. All macrographs are captured by a camera (EOS-M50, Canon, Japan). A supercontinuum fiber laser (SuperK EVO, NKT Photonics, Denmark) was filtered at different wavelengths by a multichannel acousto-optic tunable filter (SuperK SELECT, NKT Photonics, Denmark). The driving electric field was generated by a waveform generator (33500B, Keysight Technologies, USA). All light images were captured by a CMOS image sensor camera (DCC1645C, Thorlabs, USA). The electrical response curve was measured by a photodetector (PDA100A-EC, Thorlabs, USA) and an oscilloscope (MDO34, Tektronix, USA).

### Optical configuration of the dynamic two-dimentional 1st-order differentiation

As shown in Fig. 3a, the arm 1 in the Mach-Zehnder interferometer performs the differential operation in *x* direction, while the arm 2 does the differential operation in *y* direction. A couple of lenses adjacent to PBSs forms a 4 *f* system. PBS is the polarization beam splitter (PBS25-1M, JCOPTIX, China). The input is split into two orthogonally polarized components by PBS, outputs from two arms, passes through another PBS and then combines a collinear light beam. Two FLC differentiators are set at the confocal of 4 *f* system in each arm.

## Supplementary information

Supplemental Information

## Data Availability

All data supporting this study and its findings are available within this article and its Supplementary Information files. Any other relevant data are available from the corresponding authors upon request.
