# Supplementary material for: Reconfigurable ferroelectric chiral nanostructures enable fast-switchable optical spatial differentiation
Source: Light Sci Appl. 2026 Jun 26;15:285. doi: 10.1038/s41377-026-02363-w (PMC13309567; doi:10.1038/s41377-026-02363-w)
Supplement: Supplementary file 1 — Supplemental Information [file 41377_2026_2363_MOESM1_ESM.pdf]

Supplementary Information for

Reconfigurable ferroelectric chiral nanostructures enable fast-switchable optical spatial differentiation

Wen Chen, Dong Zhu, Su-Nan Chen, Yi-Heng Zhang, Si-Jia Liu, Rui Sun, Yi-Ming Wang, Lin Zhu, Shi-Hui Ding, Shi-Jun Ge, Yan-Qing Lu*, and Peng Chen*

National Laboratory of Solid State Microstructures, Key Laboratory of Intelligent Optical Sensing and Manipulation, College of Engineering and Applied Sciences, and Collaborative Innovation Center of Advanced Microstructures, Nanjing University, Nanjing 210093, China.

*E-mail: yqlu@nju.edu.cn, chenpeng@nju.edu.cn

This Supplementary Information contains two Supplementary Notes, twelve Supplementary Figures and two Supplementary Tables.

Supplementary Note 1. Fabrication process and the dynamic photopatterning technology

One bare indium-tinoxide (ITO)-coated glass substrate (1.5 × 2.0 cm^2) was subjected to ultrasonic cleaning and UV-Ozone treatment. Then, it was embedded with the photoalignment layer of SD1 by spin-coating, and cured at 100 °C for 10 min sequentially. The sulfonic azo-dye SD1 was dissolved in dimethylformamide at a concentration of 0.3 wt%. Another clear bare ITO-coated glass substrate was used to seal the cell with spacer-doped UV glue. The sandwich-like configuration was formed with desired thickness of 1.5 μm. In order to encode the expected optical axis distribution in the FLC chiral nanostructure, a dynamic photopatterning technology was adopted. One remarkable property of SD1 is the pure reorientation of molecular absorption oscillators perpendicular to UV light polarization without any photochemical transformations. Thus, the high photoalignment quality of FLCs onto SD1 layers can be achieved. Through a DMD-based dynamic micro-lithography system, UV light with varying linear polarization sequentially illuminated on different microdomains in a multi-step manner with a total dose of ~5 J·cm^{-2}. The DMD (Discovery 3000, Texas Instruments) is consisted of 1920 × 1080 micromirrors with the pixel size of 10.8 μm × 10.8 μm, and a 2× objective was used to further reduce the pixel pitch. Here, to ensure the exact distribution, we chose 180-step exposure with varying polarization 1°. As SD1 molecules reorient, the SD1 layer could be endowed with the desired alignment distribution after photoalignment process. The FLC material was injected into the photopatterned cell at 85 °C, and then gradually cooled from isotropic phase to smectic C* phase at a rate of 0.1 °C·min^{-1}. After cooling to room temperature, the FLC acquired the same optical axis distribution as SD1 through intermolecular interactions.

Supplementary Note 2. The influence of the FLC thickness

Firstly, the thickness of FLC layer influences the polarization conversion efficiency. The FLC device can be regarded as a waveplate with optical axis oriented at $\alpha(x,y)$, and its phase retardation Γ for specific wavelength depends on the FLC layer thickness d: $\Gamma = \frac{2\pi\Delta n_{\text{eff}} d}{\lambda}$, where Δn_{eff} denotes the effective birefringence, λ is the wavelength of incident light. The light modulation properties of the FLC material are determined by its retardation, and the azimuth angle α, in the form of Jones matrix:

$$J_{\text{FLC}} = \begin{bmatrix} \cos\alpha & -\sin\alpha \\ \sin\alpha & \cos\alpha \end{bmatrix} \begin{bmatrix} e^{-i\Gamma/2} & 0 \\ 0 & e^{i\Gamma/2} \end{bmatrix} \begin{bmatrix} \cos\alpha & \sin\alpha \\ -\sin\alpha & \cos\alpha \end{bmatrix}.$$

For the incidence of left-handed circularly polarized (LCP) light $|L\rangle = [1 \quad i]^T$, the modulation of right-handed circularly polarized (RCP) component in the output light can be calculated by $-i\sin(\Gamma/2)e^{i2\alpha}$. The amplitude of RCP component depends on phase retardation Γ, affecting diffraction efficiency at different wavelengths. By selecting a material with an optimal combination of thickness and birefringence, high diffraction efficiency can be attained at the operating wavelength. Here, in our experiment, the polarization conversion efficiency $\eta = \sin^2(\Gamma/2)$ is tested in Supplementary Fig. 10a. The polarization conversion efficiency of 1.54-μm-thick FLC differentiator maintains high within the 490 nm to 630 nm range.

The response time depends on the thickness of FLC layer. In ESH mode of FLCs, the response time $\tau \propto \frac{\gamma_{\varphi}}{P_S E} = \frac{\gamma_{\varphi} d}{P_S V}$, where γ_{φ} is FLC rotational viscosity, P_S is the FLC spontaneous polarization, E is applied electric field, V is the applied voltage, and d is the cell thickness. The response time is proportional to the thickness and inversely proportional to the applied voltage. Consequently, it theoretically remains constant under a uniform electric field ($E = V/d$). Keeping the applied voltage constant, a smaller thickness will be beneficial for reducing response time. But when the cell is too thin, the response time actually increases slightly, affected by strong surface anchoring. In additional experiment, the 1.54-μm-thick FLC sample performs fastest (Supplementary Fig. 5).

Also, thickness affects alignment quality, thereby affecting the imaging quality (Supplementary Fig. 6). To achieve high-quality alignment of FLC, the helix elastic energy needs to comparable but obligatory not less than the anchoring energy normalized to d. However, excessive thickness reduces the anchoring effectiveness in regions distal to the alignment surface under asymmetric boundary conditions, which degrades the overall quality.

Overall, we adopt 1.54-μm thickness, as the trade-off between imaging quality and switching speed in practical experiment.

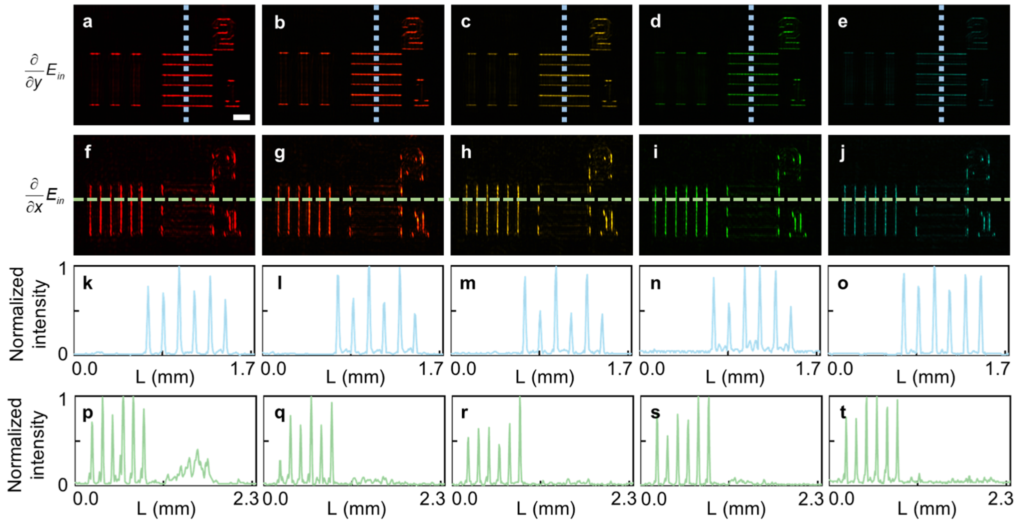

Supplementary Figure 1. First-order optical spatial differentiation in two arms of the Mach-Zehnder interferometer under +5 V. (**a**)-(**e**) 1st-order horizontal edge images, and (**f**)-(**j**) 1st-order vertical edge images at 630 nm, 600nm, 580 nm, 550 nm, 490 nm, respectively. (**k**)-(**o**) are corresponding intensity analysis of blue dotted lines marked in (**a**)-(**e**), respectively. (**p**)-(**t**) are corresponding intensity analysis of green dotted lines marked in (**f**)-(**j**), respectively. The scale bar is 200 μm.

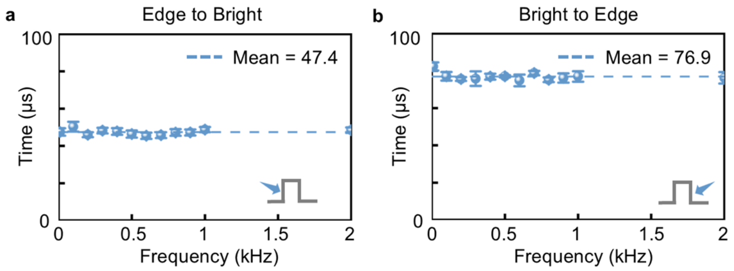

Supplementary Figure 2. Response time. (**a**) Response time from edge imaging (1st-order differentiation) to bright-field imaging. (**b**) Response time from bright-field imaging to edge imaging (1st-order differentiation). Error analysis: standard deviation based on five measurements.

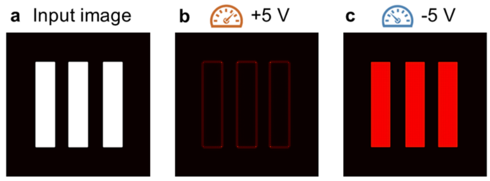

Supplementary Figure 3. Simulation results of the switchable 2nd-order optical differentiation via reconfigurable FLC superstructure. (**a**) Input image as intensity object. (**b**) The 2nd-order edge images under +5 V and (**c**) the bright-field images under -5 V.

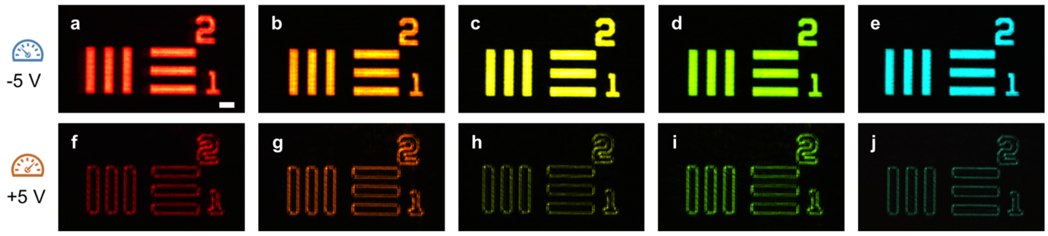

Supplementary Figure 4. Dynamically switchable 2nd-order optical spatial differentiation of intensity objects. (**a**)-(**e**) The bright images under -5 V and (**f**)-(**j**) the 2nd-order edge images under +5 V at 630 nm, 600nm, 580 nm, 550 nm, 490 nm, respectively. The scale bar is 200 μm.

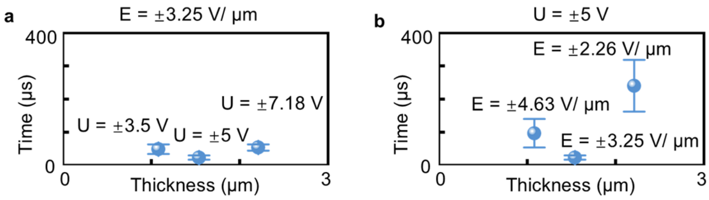

Supplementary Figure 5. Response time under different electric fields. (**a**) Imaging with different thicknesses under a constant electric field. (**b**) Imaging with different thicknesses under a constant voltage.

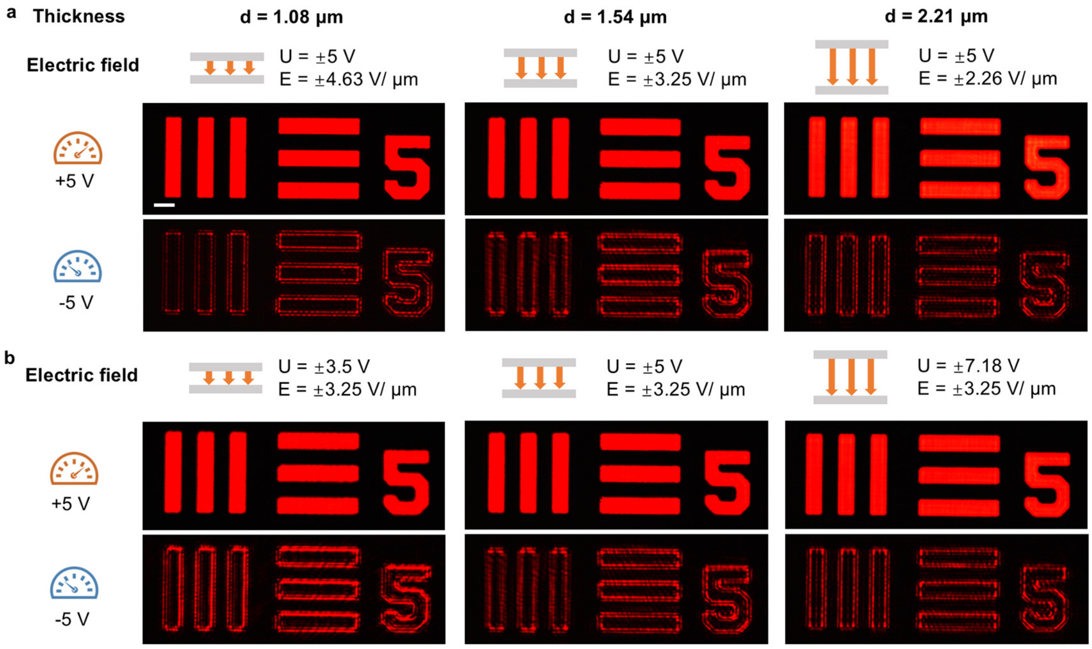

Supplementary Figure 6. Imaging under different electric fields. (**a**) Imaging with different thickness under a constant voltage. (**b**) Imaging with different thickness under a constant electric field. The scale bar is 200 μm.

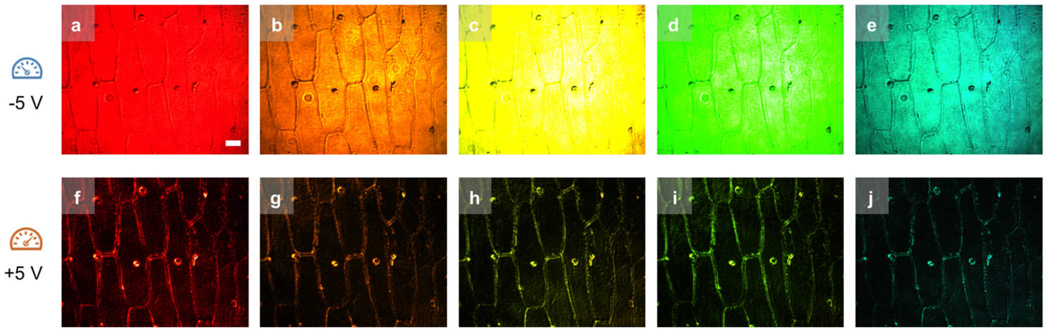

Supplementary Figure 7. Dynamically switchable 2nd-order optical spatial differentiation of phase objects. (**a**)-(**e**) The bright images under -5 V and (**f**)-(**j**) the 2nd-order edge images under +5 V at 630 nm, 600nm, 580 nm, 550 nm, 490 nm, respectively. The scale bar is 200 μm.

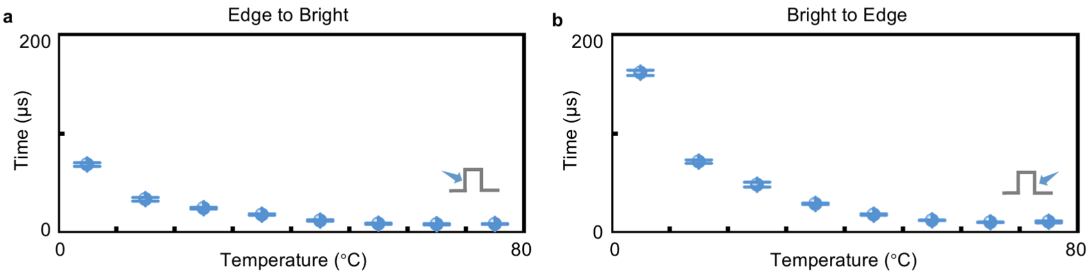

Supplementary Figure 8. Response time under different temperatures. (**a**) Response time from edge imaging to bright-field imaging. (**b**) Response time from bright-field imaging to edge imaging.

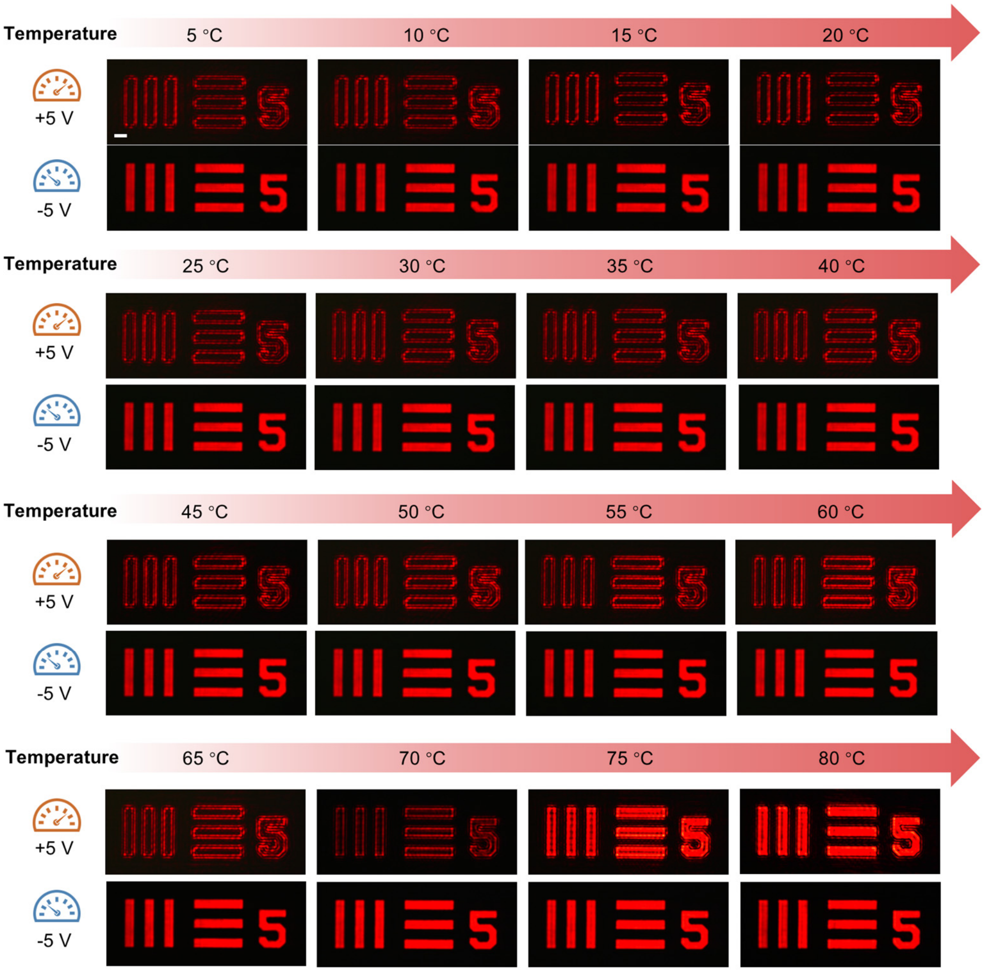

Supplementary Figure 9. Imaging under different temperatures. The FLC differentiator performs well within 0 - 70 °C, and fails to detect edges over 70 °C. The scale bar is 200 μm.

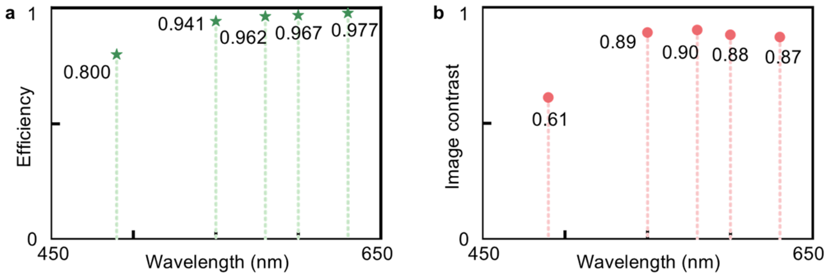

Supplementary Figure 10. Polarization conversion efficiency and image contrast under different wavelengths. (**a**) Polarization conversion efficiency and (**b**) image contrast at 490 nm, 550 nm, 580 nm, 600 nm and 630 nm, respectively.

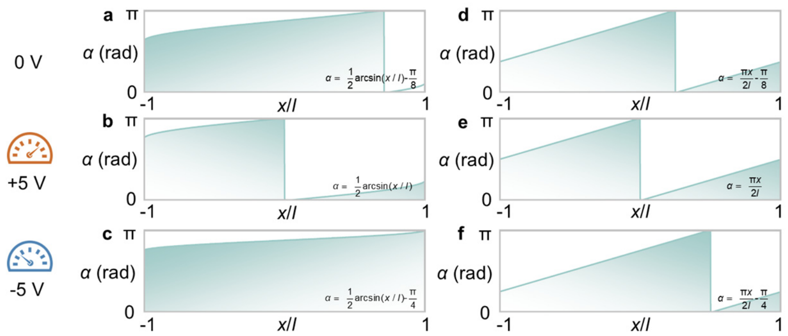

Supplementary Figure 11. Comparison of the optical axis distribution between our proposed FLC differentiator and the common FLC polarization grating (PG) along *x* axis. (**a**)-(**c**) The optical axis distribution of the FLC differentiator under 0 V, +5 V, and -5 V, respectively. (**d**)-(**f**) The optical axis distribution of the FLC PG under 0 V, +5 V, and -5 V, respectively.

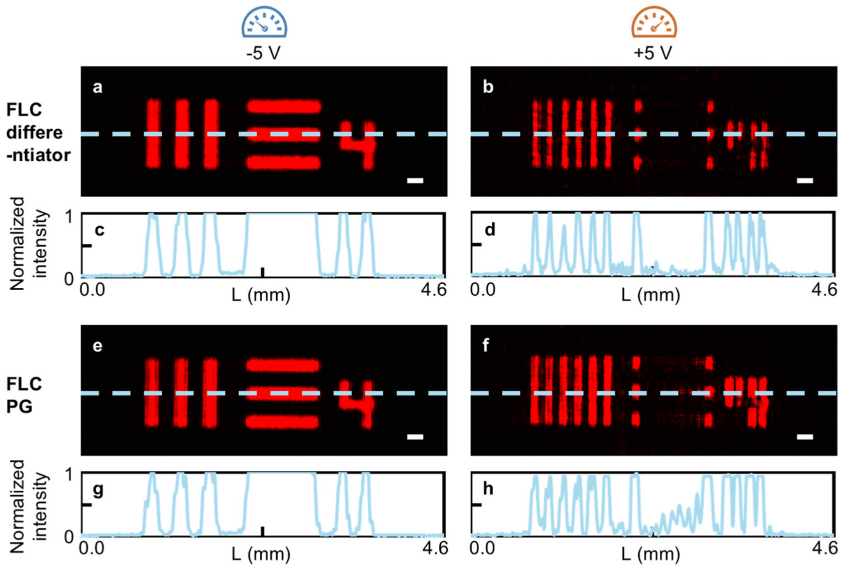

Supplementary Figure 12. Comparison of the edge imaging between our proposed FLC differentiator and the common PG along *x* coordinate. (**a**) The bright-field image of the proposed FLC differentiator under -5 V. (**b**) The edge image of the proposed FLC differentiator under +5 V. (**c**)-(**d**) are corresponding intensity analysis of blue dotted lines marked in (**a**)-(**b**), respectively. (**e**) The bright-field image of the common FLC PG under -5 V. (**f**) The edge image of common FLC PG under +5 V. (**g**)-(**h**) are corresponding intensity analysis of blue dotted lines marked in (**e**)-(**f**), respectively. All scale bars are 200 μm.

Supplementary Table 1: Comparison with existing optical differentiators

Ref	Working principle	Material	Reconfig-urability	Response time	Function	Wavelength range (nm)	Efficiency③	Resolution
[1]	Laplacian operation	VO_2	Yes	~1 s	Edge+Bright①	1670	81%	3.2 μm
[2]	Laplacian operation	Sb_2S_3	Yes	5 min	Edge+Bright	1030-1080	81%	3.5 μm
[3]	Phase contrast imaging	YVO_4	No	/	Edge	440-670	N/A④	~24.8 μm
[4]	Phase contrast imaging	α-Si	No	/	Edge	Wideband②	~80%	≥6.96 μm
[5]	Phase contrast imaging	Si	No	/	Edge	7500-13500	44%	0.272 cy/mrad
[6]	Phase contrast imaging	a-Si:H +NLC	Yes	Millisecond	Edge+Bright	Wideband	32.3%	≥7.81μm
[7]	Differential approximation	NLC	Yes	310 μs	Edge+Bright	Wideband	>95%	N/A
[8]	Differential analysis	FLC	Yes	62 μs	Edge+Bright	Wideband	>95%	~9 μm

Ref: [1] *Nat. Commun.* **15**, 4483 (2024); [2] *Light Sci. Appl.* **14**, 182 (2025); [3] *Optica* **11**, 1008-1016 (2024); [4] *Nat. Commun.* **15**, 9045 (2024); [5] *Sci. Adv.* **10**, eadk0024 (2024); [6] *ACS Nano* **17**, 14678–14685 (2023); [7] *Nanophotonics*, **13**, 327-338 (2024); [8] Our work.

① Bright: Bright-field imaging; Edge: Edge imaging.

② Wideband: not specified but suitable for wide band.

③ Peak efficiency.

④ N/A: Not Applicable.

Supplementary Table 2: Comparison with existing reconfigurable platforms

**Materials**		**PCMs**		**MEMS**	**FLCs**
		**$Ge_2Sb_2Te_5$**	**Sb_2S_3**		
**Ref**		*Nat Commun* **13**, 1696 (2022)	*Light Sci Appl* **14**, 182 (2025)	*Light Sci Appl* **8**, 59 (2019)	Our work
**Switching speed**		< 200 μs	5 min	100 μs	62 μs
**Power consumption**	**Static**	80%	81%	69%	> 95%
	**Dynamic**	250 °C/770 °C	225 °C	0-300 V	5 V/-5 V
**Volatility**		Non-volatility	Non-volatility	Volatility	Volatility
**System complexity**		Low	Low	Middle	Middle
**Functional diversity**		Beam steering-2 states	Edge imaging-2 states	Lens-N states	Edge imaging-2 states
